# Skin Directed Therapy in Cutaneous T-Cell Lymphoma

**DOI:** 10.3389/fonc.2019.00260

**Published:** 2019-04-11

**Authors:** Erica S. Tarabadkar, Michi M. Shinohara

**Affiliations:** Division of Dermatology, University of Washington, Seattle, WA, United States

**Keywords:** cutaneous T-cell lymphoma, mycosis fungoides, skin-directed therapy, nitrogen mustard, phototherapy

## Abstract

Skin directed therapies (SDTs) serve important roles in the treatment of early stage cutaneous T-cell lymphoma (CTCL)/mycosis fungoides (MF), as well as managing symptoms and improving quality of life of all stages. There are now numerous options for topical therapies that demonstrate high response rates, particularly in early/limited MF. Phototherapy retains an important role in treating MF, with increasing data supporting efficacy and long-term safety of both UVB and PUVA as well as some newer/targeted methodologies. Radiation therapy, including localized radiation and total skin electron beam therapy, continues to be a cornerstone of therapy for all stages of MF.

## Introduction

Skin directed therapies (SDTs) in cutaneous T-cell lymphoma (CTCL)/mycosis fungoides (MF) serve important roles in treating disease, but also in treating symptoms. Although SDTs can be used to cure CTCL in some patients with limited or early stage MF (stage 1A, 1B), they are most often used with palliative intent at all stages (1), with adjunct roles for both treatment and symptom management in more advanced MF, particularly managing pruritus and maintaining the skin barrier. The current National Comprehensive Cancer Network (NCCN) guidelines (1) recommend a general list of SDTs, but do not dictate the order in which they should be selected, allowing flexibility for selection based on both practitioner and patient factors.

One of the most important considerations when selecting a SDT is the extent of skin involvement (T stage). Although most SDT are appropriate for patients with any stage MF, topical preparations may be most practical (and therefore utilized with the highest compliance and best response) for those with limited skin body surface area involvement compared to patients with generalized skin involvement. In addition, a patient's ability to use a treatment as prescribed must be considered; a patient who lives alone may not be able to apply a topical therapy to their back, and someone who cannot stand without assistance may not be able to safely comply with phototherapy. Lastly, there are regional differences in preference for particular SDTs, and not all therapies are available in all regions worldwide.

Many of the studies evaluating SDTs were performed before standard definitions of clinical end points and response criteria were defined for CTCL/MF (2). As such, the difficulty comparing efficacy rates of different therapies should be considered when evaluating treatment options. A summary of the significant studies supporting the use of the various SDTs reviewed in this article is included in Table 1.

**Table 1:** Studies and treatment responses to topical therapies for CTCL.

**Treatment**	**Study**	**Study design**	**Stage**	**Response rate**	**Adverse events**
Topical steroids (class I-III)	Zackheim et al. (3)	Prospective	*n* = 79, IA and IB, patch and plaque	T1: ORR 94%, CR 63%, PR 31% T2: ORR 82%, CR 25%, PR 57%	Temporary depression of cortisol levels (*n* = 10), minor skin irritation, reversible skin atrophy
Imiquimod	Shipman et al. (4)	Case series and review	*n* = 20, IA–IIB	ORR 80%, CR 45%, PR 35%	Application site reaction, rare fatigue, flu-like symptoms
Resiquimod gel	Rook et al. (5)	Open label, phase 1 trial	*n* = 12, IA–IIA patch, plaque, folliculotropic	ORR 75%, CR 33%, PR 42%	Local skin irritation, low grade fever
Mechlorethamine solution	Vonderheid et al. (6)	Retrospective	*n* = 324, I–IV or Sezary syndrome^§^	T1 CR 80% T2 CR 62%	Allergic contact dermatitis, increased risk of cSCC
Mechlorethamine solution	Ramsay et al. (7)	Retrospective	*n* = 117, Stage I–III^*^	60.8% CR (all stages) stage I CR 75.8% stage II CR 44.6%	Delayed hypersensitivity reaction
Mechlorethamine ointment or solution	Kim et al. (8)	Retrospective	*n* = 203, T1–T4^§^	ORR 83%, CR 50%, PR 33%	Irritant or allergic contact dermatitis
Mechlorethamine	Lessin et al. (9)	Randomized, controlled, trial	*n* = 260, IA–IIA	ORR 58.5%, CR 13.8%, PR 44.6%	Skin irritation, pruritus, contact dermatitis
Carmustine	Zackheim et al. (10)	Retrospective	*n* = 143, IA–IVA^§^	IA: ORR 98%, CR 86%, PR 12% IB: ORR 84%, CR 47%, PR 37%	Mild bone marrow suppression (<10%), local skin erythema and tenderness, telangiectasia
Bexarotene gel	Breneman et al. (11)	Phase 1/2 dose escalation trial	*n* = 67, IA–IIA	ORR 63%, CR 21%, PR 42%	Local rash, pruritus, pain, rash
Bexarotene gel	Heald et al. (12)	Phase III trial	*n* = 50, IA–IIA	ORR 54%, CR 10%, PR 44%	Irritant dermatitis
Tazarotene cream	Morin et al. (13)	Open-label, prospective study	*n* = 10, IA–IIA	CR 60%	Pruritus, burning, erythema, desquamation
Tazarotene gel	Apisarnthanarax et al. (14)	Open-label pilot study	*n* = 19, Patch or plaque (<20% BSA)	ORR 58%, index lesions cleared in 35%	Skin irritation (erythema, burning, peeling)

*ORR, objective response rate; CR, complete response; PR, partial response; cSCC, cutaneous squamous cell carcinoma*.

§ *Staging according to 1978 Mycosis Fungoides*.

Cooperative Group-National Cancer Institute Workshop staging classification (15).

* *Other staging system used (7)*.

## Topical Therapies

### Topical Corticosteroids

Topical corticosteroids have been reported in CTCL since the 1960s (16). A prospective study of 79 patients demonstrated overall response rates (ORR) of 94% for T1 (IA) disease and 82% for T2 (IB) disease, with complete response (CR) rates of 63% and 25%, respectively (3). In an updated report, 200 patients with early MF were treated with class I corticosteroids, with ORR of 80–90% (17). Less potent topical steroids may also be effective when used under occlusion (18).

Cutaneous side effects to topical corticosteroids in CTCL are common. In Zackheim et al.'s series, 10–20% of patients using class I steroids for ≥3 months developed irritant dermatitis or purpura, and some developed atrophy and striae (17). Reversible suppression of cortisol levels can also occur with topical steroids in CTCL (3).

### Imidazoquinolines (Imiquimod and Resiquimod)

Imiquimod is a toll-like receptor-7 (TLR) agonist with antiviral and antitumor properties. Topical imiquimod leads to local production of interferon (IFN)-α, tumor necrosis factor-α, interleukin (IL)-1, and IL-6, (19–21) and induces direct tumor cell death and apoptosis (19, 22).

Published data on imiquimod in CTCL is limited. In a case series of 20 patients with IA-IIB mycosis fungoides (MF), treatment with imiquimod 5% yielded an ORR of 80%, with 45% CR and 35% partial response (PR). Twenty percent of patients did not respond (4). Side effects of imiquimod are typically skin-limited, including pain, redness, local irritation, ulceration, and pruritus (4). There are rare reports of patients experiencing flu like symptoms and fatigue (23, 24). Most adverse events resolve after the first few weeks of treatment, or with short treatment interruption (25, 26).

Resiquimod is a potent agonist of TLR-7 and TLR-8, leading to production of IFN-α, IL-12, and IL-15 (21). In a Phase I trial of 12 patients with IA-IIA CTCL, treatment with 0.03 and 0.06% topical resiquimod gel resulted in clinical improvement in 75% of treated lesions. Three patients had responses in untreated lesions, suggesting systemic activity. Adverse events were mostly skin limited, but 2 patients developed fever. Ninety percent of patients had a decrease in malignant T cell clones in treated lesions (5).

### Mechlorethamine Hydrochloride (Nitrogen Mustard)

Mechlorethamine hydrochloride [nitrogen mustard (NM)] is one of the most studied treatments for CTCL, with studies reporting on the topical use of NM for CTCL from the late 1950s onward (27, 28). NM is a cytotoxic alkylating agent thought to act directly on malignant cells by causing apoptosis, though the exact mechanism of action of topical NM in CTCL is unknown (22, 29).

Topical NM is a first line treatment for localized and generalized MF (1). NM can be used as a compounded solution or ointment, or a commercial gel formulation. Data on efficacy of NM vary by study and preparation, but all topical preparations have high response rates in MF, including significant rates of CR. For NM solution, 50–60% CR rates are reported (6–8). Responses are similar with aqueous solution and ointment (8). Median time to CR with NM is on the order of 6–12 months (7, 8).

The phase 2 trial leading to FDA approval of topical mechlorethamine gel (Valchlor) included 260 stage IA to IIA MF patients treated with 0.02% gel daily for up to 12 months. The ORR (CR + PR) using Composite Assessment of Index Lesion Severity (CAILS) was 58.5%, with 13.8% CR. Responses were durable; 85.5% had ongoing responses at 12 months (9).

Overall, topical NM is well tolerated, with side effects minor and skin limited (8, 9, 30). Common side effects include contact dermatitis, which is most frequent with the aqueous solution (64.7% in one series) (31). The rate of allergic contact dermatitis (ACD) with ointment preparations is significantly lower (<10%), though irritant reactions still occur in about 25% (particularly on the face and intertriginous areas). Decreasing frequency or concentration of NM may improve tolerability (8, 29). The rate of ACD with commercial NM gel was 16.4%, though 25% experienced skin irritation (9). The use of topical steroids and NM together may improve tolerability and allow for less frequent NM application (30).

The risk of secondary malignancies in patients treated with topical NM is conflicting and difficult to assess, as many patients also receive concurrent treatments (29). There are reports of MF patients with no significant risk factors developing skin cancer after topical NM (6, 32), while at least one study has showed no significant increase in secondary skin cancers with long duration of NM therapy (33).

### Carmustine

Carmustine (bis-chloroethyl-nitrosourea; BCNU) is an alkylating agent that cross-links DNA, causing apoptosis (34, 35). Zackheim reported 143 patients with CTCL treated with topical carmustine solution at variable dosing (from 2 mg/mL local up to 60 mg total body daily) (10). The ORR in patients with T1 (IA) disease was 98%, with 86% CR and 12% PR. In patients with T2 (IB) disease, ORR was 84%, with 47% CR and 37% PR. The median time to CR for all patients was 11.5 weeks (range 3–104 weeks) (10).

Cutaneous adverse events with BCNU are common, with frequent erythema, particularly in the intertriginous skin. Contact dermatitis can also occur. Telangiectasias can develop in areas of prior erythema, and may be permanent. Mild bone marrow suppression (leukopenia and anemia) can occur with widespread BCNU therapy; monthly monitoring of complete blood counts is recommended for these patients (10).

### Topical Retinoids

#### Bexarotene

Bexarotene is a retinoid X receptor agonist. Topical bexarotene 1% gel is FDA approved for the treatment of stage IA and IB persistent or refractory CTCL. The mode of action of bexarotene in CTCL is unclear, but it has been reported to cause apoptosis in CTCL cell lines (36, 37).

Topical bexarotene demonstrates benefit for early MF, however, adverse events and intolerance are common. The Phase 1/2 trial of bexarotene gel enrolled 67 patients with stage IA-IIA CTCL/MF. ORR was 63%, with CR in 21% and PR in 42%. Local adverse events occurred in 87% of patients, and included rash, pruritus, pain, and vesiculobullous rash (11). A phase III trial of topical bexarotene 1% gel showed similar findings, with an ORR of 54%, clinical CR in 10%, and frequent dose related irritant dermatitis (12).

#### Tazarotene

Tazarotenic acid binds retinoic acid receptors (RAR)-β and RAR-γ, exerting anti-proliferative and anti-inflammatory affects in the skin (38, 39). The first study of topical tazarotene as monotherapy in CTCL was published in 2016. Ten patients with early stage CTCL/MF were treated with tazarotene 0.1% cream to index lesions every other day for 2 weeks, then once daily for 6 months. Sixty percent of patients had a CR, with mean time to CR 3.8 months. Seventy percent of patients reported grade I or II side effects including pruritus, burning, erythema, and desquamation, and two patients withdrew from study because adverse events (13). Topical tazarotene 0.1% gel has also been evaluated in 19 patients as adjuvant therapy, with an ORR of 58% with once daily application for 24 weeks. Local skin irritation was reported in 84% (14).

## Phototherapy

Several groups have reviewed the existing studies and published guidelines for the use of phototherapy in CTCL/MF (40, 41). The United States Cutaneous Lymphoma Consortium (USCLC) recommends phototherapy as monotherapy for patients with early (stages IA–IIA) CTCL/MF, and in combination with systemic therapies for refractory early disease or advanced disease (41). The choice of nbUVB vs. PUVA as the initial therapy may be dictated by patient preference or by access issues. UVA has better skin penetration than UVB, and patients with thicker plaques or folliculotropic disease (41) or darker skin (42) may get more benefit from PUVA.

The USCLC offers expert consensus recommendations for phototherapy treatment protocols (41). In general, phototherapy for CTCL includes clearance, consolidation, and maintenance phases. The USCLC defines the goal of the clearance phase as 100% clearance (41). The clearance phase for CTCL may take longer than for other skin diseases that are treated with phototherapy. Patients with CTCL may also benefit from a 1–3 month long “consolidation phase” between clearance and maintenance phases, in which the frequency and dose of treatments is held constant (41). The consolidation phase may maximize the potential of histologic and molecular clearance (including loss of the dominant T-cell clone), which can lag behind clinical clearance (43, 44). Inclusion of a prolonged maintenance phase after clearance of MF may prolong the time to relapse (45) and reduce relapse rates (46), but remains controversial given the potential for increased UV exposure and a lack of significant data supporting a decrease in relapse (40).

### Contraindications and Side Effects of Phototherapy

General contraindications to phototherapy include photosensitive disorders, including xeroderma pigmentosa, lupus erythematosus, and porphyrias. PUVA should not be used in pregnant or lactating women, or those with a history of melanoma or multiple non-melanoma skin cancers. Relative contraindications to phototherapy in general include chronic actinic dermatitis and claustrophobia. Phototherapy should be used cautiously in those who are immunosuppressed, in children, or those who take photosensitizing medications (41).

Common side effects during phototherapy for CTCL include erythema and pruritus. Pruritus can be a particularly bothersome issue after starting phototherapy, particularly during PUVA (“PUVA itch”) (47). Photodamage is also a common side effect of phototherapy, particularly with PUVA, in which at least 27% of CTCL/MF patients show signs of photodamage (48).

One of the most serious potential side effects of phototherapy is secondary skin cancer. There are meta-analyses and large studies of patients assessing the risk of skin cancer with psoriasis and other skin disorders treated with UVB. In general, the risk of skin cancer overall does not appear to be significantly increased with UVB phototherapy alone (49, 50), though there may be an increase in basal cell carcinomas (BCCs) and genital skin cancers in patients who have received both UVB and PUVA (50). Patients treated with PUVA do have an increased risk of skin cancer, particularly squamous cell carcinoma (SCC) and BCC (51). In a long term study of a cohort of CTCL patients that received PUVA, the incidence of skin cancer was 26% (48).

Ultraviolet radiation induces UV-signature DNA mutations, and there is a theoretical concern that phototherapy itself could induce progression of CTCL. Hoot et al analyzed their cohort of 345 MF patients, and found that patients treated with phototherapy had a longer time to tumor progression (3.5 years) compared to those who did not receive phototherapy (1.2 years), arguing that phototherapy does not appear to increase the risk of tumor progression (52).

### Psoralen Plus UVA (PUVA)

PUVA was the first type of phototherapy used to treat CTCL (53), and is still the initial phototherapy choice preferred by many CTCL experts (54). PUVA is effective for early MF, with estimated response rates of 85% for stage IA, and 65% for stage IB (41). Patients with phototypes I or II may respond better to PUVA compared to patients with skin of color (45). Time to CR with PUVA therapy is reported in the 2–4 month range when patients are treated 2–3 times weekly (48). Those with thicker or infiltrated plaques may require longer times to clearance compared to thin plaques or patches, and PUVA is not as effective for tumor stage MF (55). Patients with hand and/or foot lesions can be treated with hand/foot bath PUVA alone or in addition to whole body treatment.

Evidence supports the use of maintenance therapy with PUVA after attaining complete clearance. Inclusion of a maintenance period is associated with longer time until relapse (45), though doesn't appear to impact overall relapse rates or survival (48).

In addition to the general contraindications and side effects of phototherapy listed above, 8-methoxypsoralen (8-MOP; methoxsalen, Oxsoralen Ultra), the most commonly used psoralen in the United States, can cause nausea and abdominal pain. 5-methoxypsoralen (5-MOP) shows similar efficacy to 8-MOP and with fewer side effects (56), but is not currently available in the United States. Psoralens can accumulate in the lens of the eye and theoretically increase the likelihood of cataracts. When eye protection is used at the current recommendation of 12–24 h after ingestion of psoralens, the risk of cataracts with PUVA therapy does not appear to be significantly increased (57).

### UVB

Broadband UVB (bbUVB) was historically used to treat CTCL/MF, with high clearance rates (71% for patients with stage IA and 44% of patients with IB), but frequent (70%) relapses (46). Among lymphoma experts, bbUVB has largely been replaced by nbUVB (54).

Narrowband UVB (nbUVB) has complete response rates in the 54–90% range (41), with patches responding better than plaques (44). Among CTCL experts, nbUVB is the initial phototherapy treatment of choice for patients with stage IA disease and fair (phototypes I and II) skin (45). Patients with the hypopigmented variant of MF may not respond as well compared to other variants of MF (42).

Whether maintenance therapy with nbUVB delays relapse is unclear. The USCLC recommends maintenance therapy given that there does seem to be decrease in relapse rate when patients undergo maintenance after complete clearance with nbUVB compared to those who do not receive maintenance (41).

In general, nbUVB is better tolerated than PUVA, with fewer reported side effects (45).

### Other Types of Phototherapy

#### Excimer (MEL)

There are several reports supporting the use of the monochromatic excimer light (308 nm) (“MEL”) for patch MF. Two series reported high response rates to MEL, with one study reporting 4/4 patients with complete clinical and histologic response (58) and another with complete clinical response in 4/5 patients (59). Follow up times were short. MEL may have a particularly useful role for sanctuary sites or sites that are not as easily accessible by phototherapy or topical preparations, such as acral surfaces (60) or intertriginous areas.

#### UVA1

Longwave or narrowband UVA (UVA1) penetrates the skin at the level of the dermis, and does not require adjunct psoralen, resulting in lower phototoxicity compared to PUVA (61). Several small series have reported on the use of UVA1 for early (stage IA–IIA) MF. The largest included 19 patients, with CR in 63% and PR in 37% with a treatment protocol of 30 J/cm^2^ 5 times a week for 5 weeks. Relapses occurred in 58% within 3 months of completing therapy (62). Prior reports have suggested higher response rates, but with low clarity on the definition of response and no follow up interval reported (63). Given low toxicity, UVA1 therapy may be a safe alternative or adjunct to other skin directed therapies.

#### Photodynamic Therapy

Photodynamic therapy (PDT) uses visible light to activate a topically applied photosensitizing agent, leading to the generation of reactive oxygen species and cell death in the affected cells. PDT demonstrates activity treating actinic keratosis and non-melanoma skin cancers (64). There are several reports supporting the use of PDT for MF. The most commonly reported photosensitizer used in MF is 5-aminolevulinic acid (ALA). In one series, plaque and tumor lesions from 10 patients were treated. Seven out of nine treated plaques had complete clinical response; tumors did not respond. A commonly reported side effect of PDT is pain; in this series, one patient dropped out because of pain, and most patients reported erythema and local edema (65).

## Radiation Therapy

MF is highly radiosensitive, and radiation therapy is effective in most stages of MF. Radiotherapy can be used with curative intent in patients with single (unilesional) early stage MF, and with palliative intent in all stages, with the goal of improving symptoms and cosmesis (66).

Electron beam (e-beam) is the preferred type of radiation therapy for MF, except in certain instances such as exophytic tumors or complex surfaces, in which X-rays or photons may be superior (67). The International Lymphoma Radiation Oncology Group (ILROG) recommends guidelines for radiation therapy for MF (67). When patients with single lesions of MF are treated with the intent to cure, CR rates can be as high as 100%, with no recurrences at treated sites (68). Radiation therapy is utilized most frequently as a palliative treatment in MF. Palliative doses of 8–12 Gy are recommended, given CR rates of >90% at doses ≥8 Gy, and higher non-response rates at lower doses (1, 69). Higher doses may be required for tumor stage or large cell transformed MF (69). For patients traveling long distances or with difficulty accessing radiation therapy, treatment can occur in a single fraction, while retaining a high CR rate (94.4%) and at significant cost savings (70). Localized radiotherapy can be especially helpful for otherwise difficult to treat sites, such as the eyelids, and the hands and feet (71).

Side effects of local radiation therapy are dose dependent, and at very low doses (2 Gy) radiotherapy to the skin can have essentially no side effects (69). Even at higher doses, side effects can be minimal (72); commonly reported side effects include erythema, desquamation, atrophy, and skin dryness (68).

Total skin electron beam (TSEBT) is a technique of delivering electron beam radiation to the entire skin surface, usually by positioning patients and exposing to multiple intersecting beams (73). TSEBT has been described as the single most effective SDT for MF (74). Traditionally, doses as high as 36 Gy (“conventional dose”) have been used, with complete response rates in the 75–95% range (73). Relapses are frequent after TSEBT, occurring in the majority of patients. Common side effects of conventional dosing TSEBT include erythema, desquamation, alopecia, nail changes, and lower extremity edema (73). Higher dose TSEBT is also associated with irreversible alopecia and low sperm counts in men (73).

Hoppe et al. published pooled data on the use of low dose (12 Gy) TSEBT for CTCL. Potential advantages to the use of low dose TSEBT over conventional dose TSEBT include lower toxicity and the potential for repeat treatment courses. ORR with low dose TSEBT was 88%, with 27% CR. Side effects were mild, reversible and less severe than reported with 36 Gy dosing. Of note, 12% of patients developed skin infections with *Staphylococcus aureus* during treatment (74); coverage with anti-staphylococcal antibiotics during TSEBT may be warranted.

The use of adjunct treatments to “consolidate” TSEBT may improve the durability. Data from the Stanford group suggest that following a TSEBT-induced CR with topical nitrogen mustard allows for longer freedom from relapse (but did not impact survival) (75). The addition of extracoroporeal photopheresis (ECP) concurrently or immediately after TSEBT has also been shown to be helpful, improving survival for patients with erythrodermic MF (76).

The literature evaluating the risk of secondary skin malignancy in CTCL is limited, but some reports suggest a possible increase in both melanoma non-melanoma skin cancer in CTCL patients with prior TSEBT (77).

## Author Contributions

All authors listed have made a substantial, direct and intellectual contribution to the work, and approved it for publication.

### Conflict of Interest Statement

MS has served as principle investigator for Actelion and Soligenix. The remaining author declares that the research was conducted in the absence of any commercial or financial relationships that could be construed as a potential conflict of interest.
